# Mitochondrial Toxicity Studied with the PBMC of Children from the Chinese National Pediatric Highly Active Antiretroviral Therapy Cohort

**DOI:** 10.1371/journal.pone.0057223

**Published:** 2013-02-27

**Authors:** Kai Liu, Yu Sun, Daojie Liu, Jiming Yin, Luxin Qiao, Ying Shi, Yaowu Dong, Ning Li, Fujie Zhang, Dexi Chen

**Affiliations:** 1 Department of Medicine, Beijing YouAn Hospital, Capital Medical University, Beijing, China; 2 Beijing Institute of Hepatology, Beijing, China; 3 Branch of Shang Cai, Henan Province, Division of Treatment and Care, National Center for AIDS/STD Control and Prevention, Henan, China; 4 Division of Treatment and Care, National Center for AIDS/STD Control and Prevention, Chinese Center for Disease Control and Prevention, Beijing, China; University of Pittsburgh, United States of America

## Abstract

As the backbone of highly active antiretroviral therapy (HAART), nucleoside reverse transcriptase inhibitors (NRTIs) have effectively improved outcomes for HIV-infected patients. However, long-term treatment with NRTIs can cause a series of pathologies associated with mitochondrial toxicity. To date, the status and mechanism of mitochondrial toxicity induced by NRTIs are still not clear, especially in HIV-infected children. As part of the national pediatric HAART program in China, our study focused on mitochondrial toxicity and its potential mechanism in HIV-1-infected children who were divided into two groups based on their duration of treatment with NRTIs: one group received treatment for less than 36 months and one group was treated for 36 to 72 months. The control group comprised age-matched non-HIV-infected children. Blood lactic acid and ATP levels in peripheral blood mononuclear cells (PBMCs) were measured to evaluate mitochondrial function, and mtDNA copies and mutations in PBMCs were determined for detecting mtDNA lesions. Simultaneously, TK2 and P53R2 gene expression in PBMC was measured. As compared with the control group, blood lactic acid levels in both NRTI treatment groups were significantly higher, whereas ATP levels and mtDNA mutation rates in PBMCs did not differ between the control and the two NRTI treatment groups. Both NRTI treatment groups exhibited significant mtDNA loss. N Moreover, we found that P53R2 mRNA expression and protein levels were significantly reduced in both treatment groups and that TK2 mRNA expression and protein levels were induced in the long-term NRTI treatment group. These results suggest that mitochondrial toxicity occurs in long-term HAART patients and that P53R2 and TK2 levels in PBMCs are useful biomarkers for detecting mitochondrial toxicity in patients on long-term treatment with NRTIs.

## Introduction

Since the clinical introduction of highly active antiretroviral therapy (HAART) in human immunodeficiency virus type 1 (HIV-1)-infected children in 1997, morbidity and mortality among these patients have improved dramatically. Nucleoside reverse transcriptase inhibitors (NRTIs) form the backbone of HAART. Long-term treatment with HAART can be associated with important adverse effects resulting from mitochondrial toxicity [1]. The primary mechanism of mitochondrial toxicity induced by NRTIs is the depletion of mitochondrial DNA (mtDNA) via the selective inhibition of DNA polymerase γ (pol γ), which is the only mitochondrial DNA polymerase for mtDNA replication and base excision repair [2]. However, the “DNA polymerase γ hypothesis” does not explain all of the effects of NRTIs on mitochondrial toxicity and is only partly responsible for various NRTI-associated adverse effects. Other mechanisms, such as oxidative damage, are assumed to be involved in NRTI toxicity. Therefore, Dr. Lewis has expanded the “DNA pol γ hypothesis” to the “mitochondrial dysfunction hypothesis,” which suggests that the mechanism of NRTI-induced mitochondrial dysfunction includes DNA pol γ inhibition, mitochondrial oxidative stress and mtDNA mutation [3].

In vitro studies with neurons and muscle and pancreatic cells have shown that NRTIs inhibit mitochondrial DNA pol γ and block mtDNA synthesis, resulting in mtDNA depletion. Different NRTIs have differential inhibitive activities on DNA pol γ. The general view is that NRTIs rank in order of mitochondrial toxicity from highest to lowest as follows: d4T and ddl > ZDV > 3TC > abacavir (ABC) and tenofovir (TDF) [4]. Studying the mechanism of mitochondrial toxicity induced by NRTIs and focusing on children with AIDS may be more urgent than focusing on adults because long-term adverse effects may have a negative impact on the children’s growth and development.

It is important to determine how to reduce the mitochondrial toxicity caused by NRTIs in HIV-1-infected neonates and children. The mechanism for how NRTI-exposed children develop symptomatic mitochondrial toxicity is complex and is affected by multiple factors, including genetic predisposition, the dose and type of NRTIs and the duration of exposure [5], [6]. Mammalian cells contain one mitochondrial nucleotide pool for mtDNA synthesis. The dNTPs in this pool are derived from the salvage of deoxyribosides catalyzed by mitochondrial kinases and from the import of deoxyribonucleotides preformed in the cytosol. NRTIs could affect advanced mitochondrial function by several mechanisms. First, NRTI monophosphates and triphosphates play a crucial role in the inhibition of DNA pol γ [7], [8]. Second, unlike nuclear DNA, mtDNA synthesis occurs not only in dividing cells but also in differentiated cells. dNTP synthesis in the mitochondrial nucleotide pool occurs via the phosphorylation of imported deoxyribonucleosides by two mitochondrial deoxyribonucleoside kinases, thymidine kinase 2 (TK2) and deoxyguanosine kinase [9]. Third, one stable R2 subunit of ribonucleotide reductase (RR) termed P53R2 has been discovered in quiescent cells, and its expression is regulated by the tumor suppressor p53 [10]. Finally, most side effects of mitochondrial toxicity can be ameliorated by changing NRTI regimens or stopping their use. These elements suggest that the mechanism of mitochondrial toxicity of NRTIs is complex and still unclear. Therefore, considering multiple factors, including virus proteins, host genetics and NRTI regimen, we should be able to identify the mechanism of mitochondrial toxicity induced by NRTIs, especially in children.

The National Pediatric HAART Program has been operating in China since 2005. To date, more than 1000 children with AIDS have been involved in this cohort. The clinical, immunologic, pharmacologic and virologic outcomes of this cohort have been reported elsewhere. This study focuses on the mitochondrial toxicity survey and potential mechanisms. First, blood lactic acid and ATP levels were measured to evaluate mitochondrial function in these patients. Then, mtDNA copies and mutations in PBMCs were assessed to detect mtDNA lesions. Finally, we quantified TK2 and P53R2 gene expression in PBMCs. Our results suggest that mitochondrial toxicity is present in long-term HAART patients and that P53R2 expression in PBMCs is a useful biomarker for detecting mitochondrial toxicity in HAART.

## Materials and Methods

### Subjects and Sample Collection

A total of 152 children (median age, 10 years; age range, 2 to 16 years) who regularly received HAART treatment from July 2005 to December 2009 were recruited into our study; these children were also a subset of the Pediatric AIDS Clinical Trial Group (PACTG) from the Centers for Disease Control of Henan Province. Our previous study had described the detailed information about these patients [11]. According to the guidance for AIDS diagnosis and treatment published by the Ministry of Health in 2005, all of the children were HIV-1-positive by enzyme-linked immunosorbent assay (ELISA) screening and western blot test (WB) confirmation and in need of antiretroviral therapy. The HAART-negative control group (Group A) comprised 50 children from the normal health survey in Beijing. The average age was 11 (4 to 16) years old, and the ratio of boys to girls was 1.5 (30/20). The PBMC from 20 cases of age-matched, untreated, HIV-infected children were obtained from our HIV blood samples bank and the total DNA in these samples were isolated for mtDNA loss specific assay.

### Primary Reagents and Instruments

QIAamp DNA Blood Mini Kit, QIAamp RNA Blood Mini Kit (QIAGEN Inc., Germany); SuperScript III First-Strand Synthesis System (Invitrogen Inc., U.S.); 10× PCR buffer, 2.5 nM dNTP, Taq DNA polymerase, 10× DNA loading buffer, DNA marker (TaKaRa Inc., Japan); lactic acid detection kit (RANDOX Inc., UK); PCR Mastercycler (Eppendorf Inc., Germany); DYY-6C electrophoresis apparatus (Bio-Rad Inc., U.S.); 1600R gel imager (Tanon Inc., China); Au5400 automatic biochemical analyzer (Olympus Inc., Japan); AutoLumat LB 953 automatic tube luminometer (BERTHOLD Inc., Germany); 7900HT Real-Time Quantitative PCR system (ABI Inc., U.S.); and PCR primers and probes were synthesized by Invitrogen.

### Blood Lactic Acid Detection

The reagents were added into standard and fresh plasma samples, mixed and incubated at 37°C for 5 minutes. The absorbance of standard and plasma samples was detected within 30 minutes. Then, the lactate levels in plasma samples (normal range of 0.5–2.22 mmol/L) were calculated according to standards.

### ATP Detection in PBMC

Peripheral blood mononuclear cell (PBMC) was isolated from fresh whole blood. A total of 1×10^4^ PBMCs was resuspended in 40 µl of lysis buffer (25 mM K_2_HPO_4_/KH_2_PO_4_ buffer, pH 7.8, 10% glycerol, 1% Triton X-100, 1 mg/ml BSA, 2 mM EGTA and 2 mM DTT), frozen in liquid nitrogen and thawed 3 times and centrifuged for 10 minutes (15000 g, 4°C). The supernatant was placed in an automatic tube luminometer for ATP analysis.

### DNA Isolation and Quantitative Detection of Mitochondrial DNA

Total DNA in PBMCs was extracted using the QIAamp DNA Blood Mini Kit following the manufacturer’s instructions. Relative mtDNA copy numbers were measured by a quantitative real-time polymerase chain reaction (qPCR) assay as described previously [12]. Briefly, the mtDNA copy number gene was cytochrome C oxidase II, and the reference gene was GAPDH. The 2X Mix (ABI Inc., USA), primers, probes (table 1) and DNA templates were combined in a 20 µl PCR reaction. The thermal cycling conditions were as follows: 2 minutes at 50°C and 1 minute at 95°C to activate the hot-start Taq DNA polymerase, followed by 40 cycles consisting of a 15 s denaturation at 95°C and a 40 s anneal/extension stage at 60°C.

**Table 1:** Primers and probes for real-time qPCR and D-loop PCR.

COX II	Sense Primer	5′-CCCCACATTAGGCTT AAAA ACAGAT-3′
	Anti-Sense Primer	5′-TATACCCCCGGTCGTGTAGCGGT-3′
	probe	5′-FAM-CAATTCCCGGACGTCTAAACCAAACCACTTTC -TAMRA-3′
GAPDH	Sense Primer	5′-CGGGGCTCTCCAGAACATC-3′
	Anti-Sense Primer	5′-ATGACCTTGCCCACAGCCT-3′
	probe	5′-FAM-CCCTGCCTCTACTGGCGCTGCC-TAMRA-3′
TK2	Sense Primer	5′-GAAGAGATGCAGGGAAGAGGAGAAG-3′
	Anti-Sense Primer	5′-GCCACTCCTCATGGAGATGGTGAAT-3′
	probe	5′-FAM-TCATTCCGCTGGAATACCTGGAAGC-TAMRA-3′
P53R2	Sense Primer	5′-AGGCACAGGCTTCCTTCTGGACAGCA-3′
	Anti-Sense Primer	5′-CATCTGCTTTAAGCTTGTTCCAGTG-3′
	probe	5′-FAM-AGAGGTCGACTTATCAAAGGATCTCCC-TAMRA-3′
D-loop	Sense primer	5′-AAAGTCTTTAACTCCACC-3′
	Anti-Sense primer	5′-CTAAGAGCTAATAGAAAGGCTAGGAC-3′

### Total RNA Isolation from PBMCs and TK2 and P53R2 mRNA Detection by qRT-PCR

RNA was extracted from PBMCs using the QIAamp RNA Blood Mini Kit following the manufacturer’s instructions. The RT step was performed using the SuperScript III RT kit (Invitrogen) following the standard protocol. TK2 and P53R2 transcript levels were also measured using a quantitative real-time reverse transcription PCR (qRT-PCR) assay. The reference gene was GAPDH. The probes and primers used for TK2 and P53R2 detection are listed in table 1. The PCR conditions were similar to those used for the mtDNA detection described above.

### D-loop and COX II Mutation Assay Using PCR and DNA Sequencing

A total of 1 µg of DNA template was added to a 50 µL PCR reaction. The thermal cycling conditions were as follows: 5 minutes at 94°C to pre-denature the templates, followed by 40 cycles consisting of a 30 s denaturation at 94°C, a 30 s annealing step at 55°C and a 30 s extension at 72°C. The PCR products were extracted and sequenced by Biomed, Inc. (Beijing, China). The PCR primer sequences (also the sequencing primers) are shown in table 1. Then, BLAST analysis was performed on the sequenced D-loop and COX II mtDNA using BioEdit software and referenced to the Cambridge mtDNA sequence (NC_012920).

### Western Blotting

Western blot analysis was performed to monitor TK2, P53R2, COX II, COX IV and tubulin protein levels in PBMC lysates. The western blotting method has been described previously. Briefly, the cells were lysed in high salt lysis buffer (150 mM NaCl, 1% NP-40, 0.5% deoxycholate, 0.1% SDS, 50 mM Tris [pH 8.0], 5 mM EDTA), the protein content was estimated by the BCA method, and 50 µg of protein was electrophoresed on a 10% SDS-PAGE gel. The blot was incubated with primary antibodies at 4°C overnight, and the membranes were visualized using a horseradish peroxidase-conjugated secondary antibody and a chemiluminescent detection system (Jinshan Inc., Beijing, China).

### Statistical Analysis

All data were processed with SPSS11.5 (Chicago). Measurement data are presented as the mean ± standard deviation. The differences between groups were assessed by an independent sample t-test. P<0.05 indicates that a difference is statistically significant.

## Results

### Patient Information

Our study focused on mitochondrial toxicity among the 152 children with AIDS in the Chinese National Pediatric Highly Active Antiretroviral Therapy Cohort, and data are shown in table 2. All patients were from Shangcai County in Henan Province and were a subset of the Pediatric AIDS Clinical ARV Trial Group of the CDC of China. The CD4 levels of all patients were less than 350 cells/µl when they were enrolled in the Pediatric AIDS Clinical ARV Trial Group. The patients were placed into two groups depending on the initiation of HAART. The group of patients who had received HAART for less than 36 months included 68 children with AIDS (Group B). The average age in this group was 8 years old (from 2 to 12 years), and the gender ratio was 35∶33 (male: female). In this group, 82% of the children were infected via mother-to-child transmission and 15% were infected through blood. Among these children, 66% were treated with d4T+3TC, 28% with AZT+3TC, 4.5% with d4T+ddI and 1.5% with AZT+ddI. The group of patients who had received HAART from 36 to 72 months included 84 children with AIDS (Group C). The average age of these children was 13 years old (from 7 to 16 years) and the gender ratio was 49∶35 (male:female). In this group, 81% of children were infected via mother-to-child transmission and 16.6% were infected through blood. HAART included d4T+3TC for 54% of these children, AZT+3TC for 39%, d4T+ddI for 3.5% and AZT+ddI for 3.5%.

**Table 2:** Patient information.

Duration of therapy	Age (years)	Gender		Regimens of NRTIs+PIs				Transmission		
		M	F	D4T+3TC +NVPor NFV	AZT+3TC NVPor NFV	D4T+ddI NVPor NFV	AZT+ddI NVPor NFV	MTCT	Blood	N/A
ControlGroup A	11 (4–16)	30	20							
<36 MGroup B	8 (2–12)	35	33	45	19	3	1	56	10	2
36–72 M Group C	13 (7–16)	49	35	45	33	3	3	68	14	2
Total		84	68	90	52	6	4	124	24	4

### Plasma Lactate Levels were Increased and No Significant Difference in ATP Levels in PBMCs was Observed Following Longer-term Treatment with NRTIs

**Figure 1:**
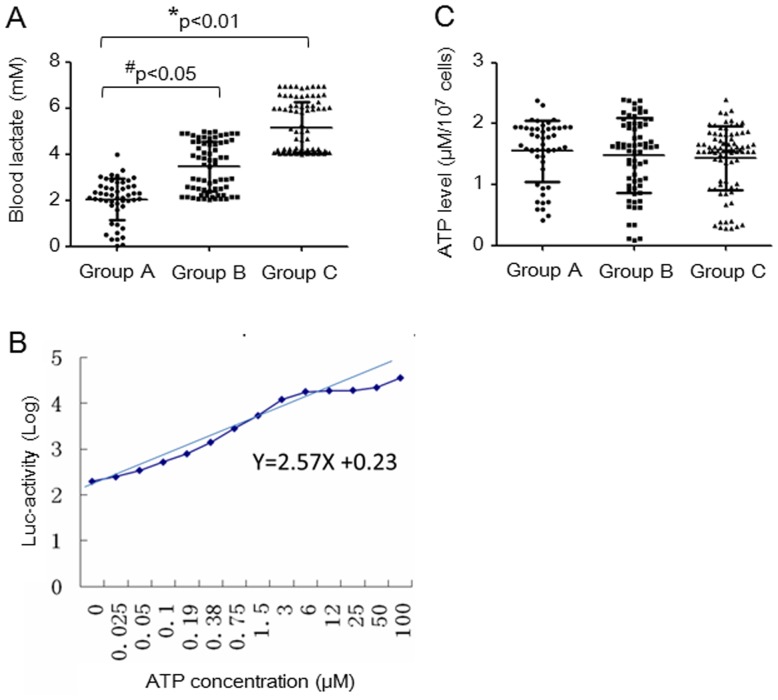
Plasma lactate and PBMC ATP levels among control and NRTI-treated children with AIDS. A: The concentration of plasma lactate in control children (Group A, n = 50),children with AIDS treated for less than 36 months (Group B, n = 68) and children with AIDS treated for 36 to 72 months (Group C, n = 84); *P<0.01, ^#^p<0.05. B: Linear relationship between the log of luciferase activity and ATP concentration. C: PBMC ATP levels in control children (Group A, n = 50), children with AIDS treated for less than 36 months (Group B, n = 68) and children with AIDS treated for 36 to 72 months (Group C, n = 84).

Previous studies have suggested that blood lactate levels are a useful biomarker for mitochondrial dysfunction, especially for severe cases [13]. The duration of antiretroviral treatment ranged from 6 to 72 months in this study, so it was important for us to determine the association between blood lactate levels and duration of treatment with NRTIs. A total of 202 samples, including 152 children treated with antiretrovirals (ARVs) and 50 control children, were analyzed in this study. The results are shown in figure 1A. We found that plasma lactate levels were 2.1±0.93 (mM) in the control group, 3.46±1.08 (mM) in the group receiving ARV treatment for less than 36 months, and 5.14±1.16 (mM) in the group treated with ARVs for 36 to 72 months. Statistical analysis revealed that, compared with the control group, lactic acid levels were significantly higher in both of the groups mentioned above. The t-test revealed a significant difference between the group with an ARV treatment duration of less than 36 months and the control group (p<0.05) and between the group with an ARV treatment duration of 36 to 72 months and the control group (p<0.01). This observation suggests that the increase in plasma lactate levels is associated with the duration of ARV treatment. Because blood lactate levels are an indicator of mitochondrial function, these results suggest that children with AIDS in this cohort on long-term HAART could suffer severe mitochondrial injury.

Mitochondria are the energy factories of cells, and cellular ATP is mainly produced by mitochondrial oxidative phosphorylation (OXPHOS). Depletion and mutation of mtDNA may reduce the efficiency of OXPHOS and result in a reduction in ATP production. When the quantities of ATP cannot maintain the activities of cells, cells enter into apoptosis [14]. Therefore, we investigated ATP levels in PBMCs to determine whether they were attenuated due to the mitochondrial toxicity induced by antiretroviral treatment. We first used an ATP standard to calculate the relevant curve between luciferase activity and ATP concentration. Our results show that this relationship is a typical S-type curve (fig. 1B). After log-transforming the luciferase values, there is a linear relationship between luciferase emission and ATP concentration. (Fig. 1B). The equation for the linear regression curve was Y = 2.57X+0.23. We compared ATP levels in the PBMCs from the three groups (Fig. 1C). Our results show that ATP levels were 1.55±0.05 in the control group, 1.48±0.05 in the group treated for less than 36 months and 1.43±0.06 in the group treated for 36 to 72 months. There was no significant difference in the ATP levels among the group treated with ARVs for less than 36 months, the group treated for 36 to 72 months and the control group according to the t-test.

### mtDNA Mutation and Depletion in PBMCs Following Longer-term Treatment with NRTIs

**Figure 2:**
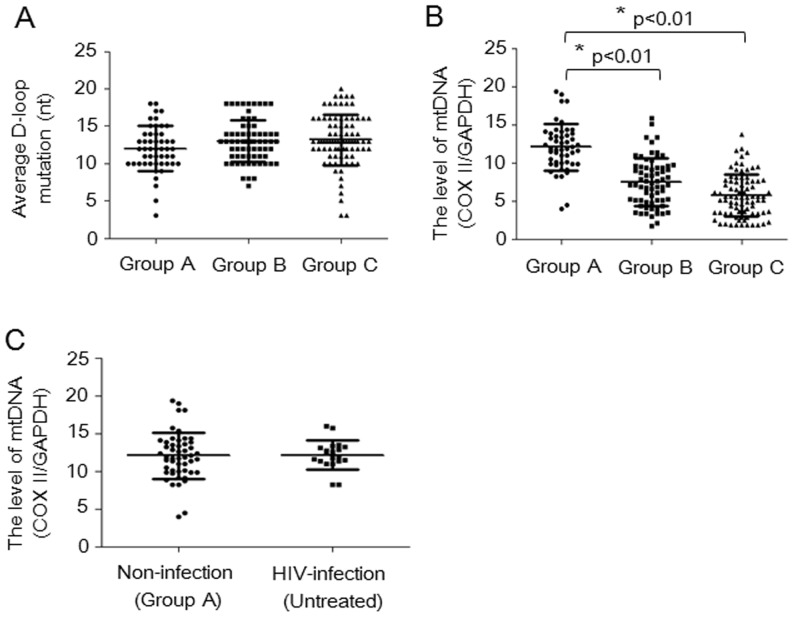
mtDNA D-loop mutations and mtDNA depletion among control and NRTI-treated children with AIDS. A: The ratio of mtDNA D-loop mutations in the control children (Group A, n = 50), children with AIDS treated for less than 36 months (Group B, n = 68) and children with AIDS treated for 36 to 72 months (Group C, n = 84). B: mtDNA depletion in control children (Group A, n = 50), children with AIDS treated for less than 36 months (Group B, n = 68) and children with AIDS treated for 36 to 72 months (Group C, n = 84); *P<0.01. C: mtDNA depletion in non-infected children (Group A) and 20 cases of age-matched, untreated, HIV-infected children (HIV-infection, n = 20), who came from our HIV blood samples bank and the total DNA in these samples were isolated for mtDNA loss specific assay.

Mitochondrial toxicity during long-term treatment with NRTIs is caused by the inhibition of DNA pol γ, which plays a crucial role in mtDNA replication and repair, resulting in mtDNA depletion and mutation. A previous study showed that the noncoding region of the mtDNA displacement loop (D-loop) contains two hypervariable regions (HV1 at positions 16024–16383 and HV2 at positions 57–372). In this study, we identified a relationship between the duration of exposure to NRTIs and mitochondrial D-loop mutations in PBMCs among children with AIDS by studying the HV2 region of the mitochondrial D-loop sequence. Our results show that the average rate of mutations was 12±3 nt in the 50 control children, 13±2.8 nt in the 68 children with AIDS treated for less than 36 months and 13±3.4 nt in the 84 children with AIDS treated for 36 to 72 months (fig. 2A). The nucleotide mutation rate did not differ among controls and the two NRTI-treated groups. These results suggest that the rate of HV2 mutation in children with AIDS treated with NRTIs is not associated with the duration of treatment.

Mitochondrial DNA depletion is the main source of mitochondrial toxicity. The side effect of mitochondrial toxicity induced by comprehensive HIV antiviral therapy causes a reduction in cell energy supplies and apoptosis. Therefore, mtDNA levels reflect the severity of mitochondrial toxicity induced by NRTIs. In previous studies, We and Mambo et al. used real-time quantitative PCR to analyze mtDNA integrity, damage repair, and induced mutations after exposure of cells to 4-nitroquinoline 1-oxide [12], [15], and Bai et al. used quantitative PCR analysis to study mtDNA content in patients with mitochondrial disease [16]. In these studies, GAPDH and 18S rRNA were used as standard quantitative PCR controls. Therefore, we analyzed endogenous mtDNA depletion with GAPDH as a standard quantitative PCR control. mtDNA levels were quantified using real-time qPCR to detect the ratio of the mtDNA COX II gene and the genomic housekeeping gene GAPDH in PBMCs. The results are shown in Figure 2B. We found that mtDNA levels in PBMCs were significantly different among the control group, the group treated with ARVs for less than 36 months and the group treated with ARVs for 36 to 72 months. Our results suggest that mtDNA loss is associated with the duration of treatment with NRTIs among children with AIDS. To determine whether the findings in figure 2B are specific for antiretroviral therapy or due to HIV infection, we compared PBMC mtDNA copies between age-matched, untreated, HIV-infected children (n = 20) and non-HIV-infected children (group A) (figure 2C). Our results show that mtDNA copy numbers between age-matched, untreated, HIV-infected children and non-HIV-infected children do not differ. These results suggest that mtDNA loss in the PBMCs from this cohort is specific to antiretroviral therapy.

### Longer-term Treatment with NRTIs can Induce TK2 Overexpression in PBMCs

**Figure 3:**
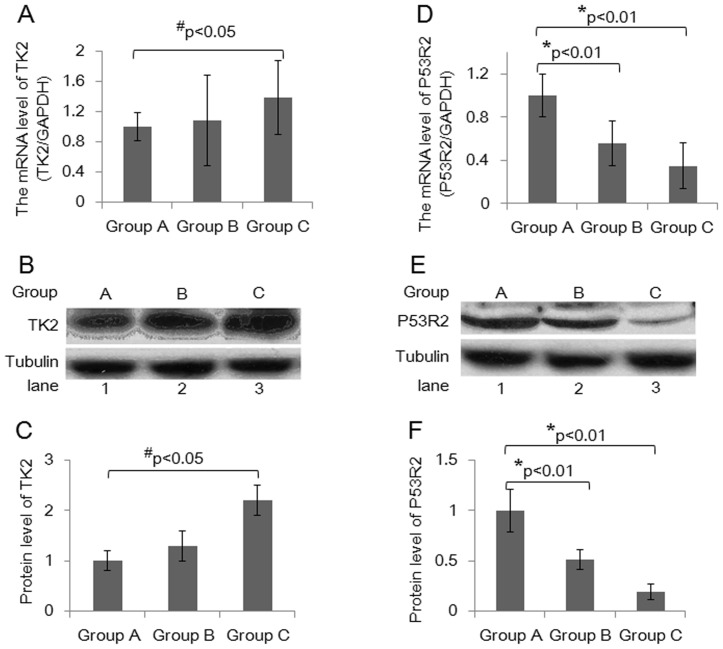
Long-term treatment with NRTIs can induce TK2 overexpression and reduce P53R2 expression in PBMCs. A: TK2 mRNA expression was quantified by qRT-PCR in control children (Group A, n = 50), children with AIDS treated for less than 36 months (Group B, n = 68) and children with AIDS treated for 36 to 72 months (Group C, n = 84); ^#^P<0.05. B: Western blot of TK protein expression in Group A, Group B and Group C. C: The graphics from 3B are shown in 3C. Data from triplicate independent experiments are presented as the mean ± SD. ^#^P<0.05. D: P53R2 mRNA expression was quantified by qRT-PCR in control children (Group A, n = 50), children with AIDS treated for less than 36 months (Group B, n = 68) and children with AIDS treated for 36 to 72 months (Group C, n = 84); *P<0.01. E: Western blot of P53R2 protein expression in group A, group B and group C. F: The graphics from 4B are shown in 4C. Data from triplicate independent experiments are presented as the mean ± SD. *P<0.01.

Thymidine kinase 2 (TK2), one of the deoxynucleoside kinases (dNKs) in humans, plays a critical role in the initial phosphorylation of pyrimidine nucleosides in the salvage pathway in mitochondria [9]. Pyrimidine nucleoside analogues (AZT, d4T and 3TC) must be phosphorylated into diphosphate or triphosphate forms by TK2 in the mitochondria or by TK1 in the cytoplasm. We analyzed TK2 transcription levels in PBMCs using a real-time quantitative RT-PCR assay. The results are shown in figure 3A. TK2 transcription levels in PBMCs did not differ between the group of children with AIDS treated for less than 36 months and the control group. However, in the group of children with AIDS treated for 36 to 72 months, TK2 mRNA levels were significantly induced. TK2 levels were 3-fold higher in the group of children with AIDS treated for less than 36 months as compared with control PBMCs. To confirm whether TK2 protein expression was consistent with mRNA levels, we randomly divided the samples in each group into 3 subgroups and prepared protein to assess TK2 protein by western blot assay in three independent experiments. Our results show that, compared with the control group, the concentration of TK2 protein was 2.2-fold higher in the group of children with AIDS treated for 36 to 72 months (Figure 3B and 3C), suggesting that TK2 induction in the PBMCs of children with AIDS is associated with long-term HAART.

### P53R2 Expression is Significantly Reduced in PBMCs from HIV-infected Children Following Treatment with NRTIs

P53R2, which is induced by the tumor suppressor p53, has recently been shown to be an important cause of mitochondrial-associated inherited human diseases [17], [18]. Research has shown that P53R2 is specifically expressed in non-cycling cells. As terminally developed blood cells, most PBMCs are non-cycling and short lived. Study of P53R2 expression in PBMC cells would be useful for understanding mitochondrial toxicity. Using quantitative RT-PCR (qRT-PCR), we analyzed P53R2 expression under different durations of NRTI exposure (Figure 3D). We found an approximately 44% reduction in P53R2 levels in the group of children with AIDS treated for less than 36 months and a nearly 65% reduction in the group treated for 36 to 72 months compared with the control group. To confirm whether P53R2 protein expression was consistent with mRNA expression, we randomly divided the samples in each group into 3 subgroups and prepared protein to assess P53R2 protein levels by western blot assay in three independent experiments. Our results show that, compared with the control group, the concentration of P53R2 protein was 2-fold lower in the group of children with AIDS treated for less than 36 months and 12-fold lower in the group treated for 36 to 72 months (Figure 3E and 3F). These results suggest that the reduction in P53R2 parallels mtDNA deletion in HIV-infected children following HAART.

### mtDNA Loss Parallels the Decrease in P53R2 Levels

**Figure 4:**
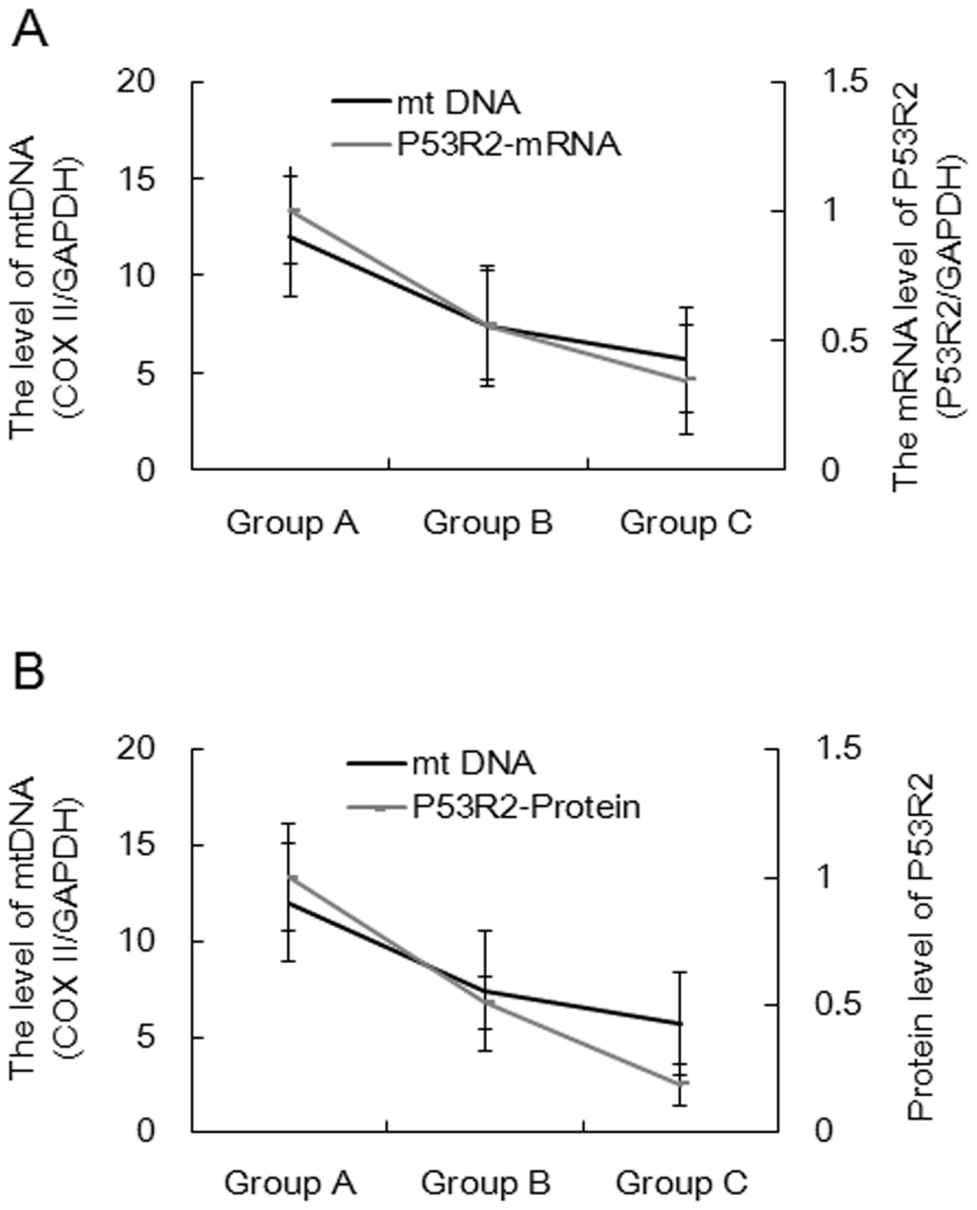
P53R2 reduction parallels mtDNA depletion in PBMCs during longer-term treatment with NRTIs. A: The association between P53R2 mRNA expression and mtDNA copy number in Groups A, B and C. B: The association between P53R2 protein expression and mtDNA copy number in Groups A, B and C.

The results above demonstrate that P53R2 expression in PBMCs is affected by HAART. Next, we wanted to determine whether P53R2 mRNA and protein levels are associated with mtDNA loss (figure 4A, mRNA level and mtDNA loss curve; figure 4B, protein level and mtDNA loss curve). These results show that mtDNA loss parallels P53R2 reduction at both the mRNA and the protein level, suggesting that P53R2 is a valuable mitochondrial toxicity biomarker in PBMCs.

## Discussion

Mitochondrial toxicity in AIDS patients caused by HAART is a concern worldwide. Because mitochondrial toxicity is associated with the type, dose and consistency of NRTIs, clinical manifestations are complex and diverse [19]–[22]. Severe mitochondrial toxicity is particularly evident in non-dividing cells, such as nerve, cardiac and skeletal muscle cells.

The primary mechanism of mitochondrial toxicity induced by NRTIs is the depletion of mitochondrial DNA (mtDNA) by the selective inhibition of DNA polymerase γ [23], which is the only DNA polymerase in mitochondria for replication and base excision repair (BER) of mtDNA. The best method for diagnosing mitochondrial toxicity induced by NRTIs is examining biopsy materials from high mitochondria copy number tissues, such as muscle, liver, or neural tissue; however, the collection of these biopsy specimens is not practical, especially for vulnerable children. Therefore, many previous studies of mitochondrial toxicity in children treated with HAART use PBMCs. One European cross-sectional analysis of 477 HIV-1-infected children (average age 10 years old) and HAART treatment administered for more than four years and six months showed that 1/4 of the children had abnormal peripheral and/or central fat distribution and 1/3 had dyslipidemia. This study also demonstrated that the apoptosis and mitochondrial dysfunction induced by NRTIs in PBMCs were significantly different in these children and independent of abnormal fat metabolism [24]. Another research team reported that mitochondrial dysfunction and apoptosis induced by NRTIs in the PBMCs of children did not differ from that of the normal control group [25]. PBMCs are easily obtained from patients; however, the attribution of morbidity and mortality to HAART-related mitochondrial toxicity is controversial, which may be due to research methods and the tissue-specific nature of mitochondrial diseases. Therefore, more research on novel biomarkers is required to elucidate the importance of mitochondrial depletion in PBMCs in the clinical setting, especially in children for whom the levels of sample materials are limited.

The longest duration of NRTI administration to children with AIDS in the Chinese National Pediatric Highly Active Antiretroviral Therapy Cohort was six years. At present, five different durations of ARV in this cohort are in progress, from ten months to six years. More than 93% of regimens administered are d4T+3TC+NVP (EFV) or AZT+3TC+NVP (EFV). Additionally, more than 63% of children with AIDS in this cohort have been treated with d4T. A previous study showed that d4T and AZT have strong side effects associated with mitochondrial toxicity [26], [27]. One study showed that mitochondrial toxicity is associated with the duration of treatment with NRTIs [28]. However, although there are five treatment groups with treatment periods ranging from ten months to six years, it is difficult to reach statistical significance because there are fewer than 30 patients in each group. Therefore, we opted to divide the patients into two groups. One group had less than 36 months of NRTI exposure (68 children) and the other group was treated for 36 to 72 months (84 children). Then, we explored mitochondrial toxicity in the PBMCs and serum of these children to identify potential mitochondrial toxicity biomarkers. In our cohort study, we found that blood lactate levels were more than two and four times higher in the group of children with AIDS treated for less than 36 months and the group treated for 36–72 months, respectively, than the control group. We also found a significant loss in mtDNA among children with AIDS exposed to NRTIs from 9 to 72 months. These results are consistent with previous reports that increased blood lactate levels and mtDNA loss in PBMCs are associated with duration of NRTI exposure. These markers are potentially useful assays for mitochondrial toxicity in children with AIDS with long-term exposure to HAART and are the standard control for identifying other potentially useful mitochondrial toxicity biomarkers.

Recent studies have shown that P53R2, the ribosomal protein regulated by p53, is involved in the repair of nuclear DNA and replication of mtDNA [29]. Once this gene has lost function, the genetic mutation familial genetic cardiomyopathy will occur, which is mainly caused by the loss of mitochondria [17]. A recent study found that mutation of P53R2 can cause human inherited diseases associated with the depletion of mitochondria that arises in the skeletal muscle of patients. Similar results were found in P53R2-knockout mice, which exhibited a near total loss of mtDNA. Therefore, P53R2 expression levels will directly affect mitochondrial function. Our results show that P53R2 expression levels in PBMCs from both the short-term and the long-term NRTI treatment groups were significantly reduced. A highly significant observation was that decreased P53R2 expression parallels mtDNA loss (Figure 4). This observation suggests that P53R2 reduction in PBMCs during treatment with NRTIs is a relevant biomarker for mitochondrial toxicity.

TK2 is primarily localized to mitochondria, and its main function is to convert monophosphate pyrimidine nucleotides to triphosphate pyrimidine nucleotides, which are the raw materials for the synthesis of mtDNA [9]. AZT, d4T and 3TC are all pyrimidine nucleoside analogues that can also be phosphorylated into triphosphates by TK2 in the mitochondria and thereby inhibit pol γ activity [30]. Point mutations in the gene encoding TK2 are correlated with mtDNA disorders in a heterogeneous group of patients suffering from the so-called mtDNA depletion syndrome (MDS), which is a syndrome with symptoms resembling those of AIDS patients treated with nucleoside analogues, but the mechanisms behind these mitochondrial effects are still not well understood [31]–[33].

In this study, we found that TK2 was overexpressed in the group with long-term exposure to NRTIs. Depending on the kinetic principle, one explanation could be that the long-term positive feedback by high levels of AZT, d4T and 3TC substrates accelerates the process of mitochondrial toxicity. The detailed mechanism and toxic effects require further investigation.

## References

[1] Pinti M, Salomoni P, Cossarizza A (2006) Anti-HIV drugs and the mitochondria. Biochim Biophys Acta 1757: 700–707. 16782042 10.1016/j.bbabio.2006.05.001

[2] Lewis W, Dalakas MC (1995) Mitochondrial toxicity of antiviral drugs. Nat Med 1: 417–422. 7585087 10.1038/nm0595-417

[3] Lewis W, Copeland WC, Day BJ (2001) Mitochondrial dna depletion, oxidative stress, and mutation: mechanisms of dysfunction from nucleoside reverse transcriptase inhibitors. Lab Invest 81: 777–790. 11406640 10.1038/labinvest.3780288

[4] Kakuda TN (2000) Pharmacology of nucleoside and nucleotide reverse transcriptase inhibitor-induced mitochondrial toxicity. Clin Ther 22: 685–708. 10929917 10.1016/S0149-2918(00)90004-3

[5] Kohler JJ, Lewis W (2007) A brief overview of mechanisms of mitochondrial toxicity from NRTIs. Environ Mol Mutagen 48: 166–172. 16758472 10.1002/em.20223

[6] Lo JC, Kazemi MR, Hsue PY, Martin JN, Deeks SG, et al (2005) The relationship between nucleoside analogue treatment duration, insulin resistance, and fasting arterialized lactate level in patients with HIV infection. Clin Infect Dis 41: 1335–1340. 16206112 10.1086/496981

[7] Eriksson S, Xu B, Clayton DA (1995) Efficient incorporation of anti-HIV deoxynucleotides by recombinant yeast mitochondrial DNA polymerase. J Biol Chem 270: 18929–18934. 7642550 10.1074/jbc.270.32.18929

[8] Lewis W, Day BJ, Copeland WC (2003) Copeland, Mitochondrial toxicity of NRTI antiviral drugs: an integrated cellular perspective. Nat Rev Drug Discov 2: 812–822. 14526384 10.1038/nrd1201

[9] Rampazzo C, Fabris S, Franzolin E, Crovatto K, Frangini M, et al (2007) Mitochondrial thymidine kinase and the enzymatic network regulating thymidine triphosphate pools in cultured human cells. J Biol Chem 282: 34758–34769. 17913703 10.1074/jbc.M705923200

[10] Tanaka H, Arakawa H, Yamaguchi T, Shiraishi K, Fukuda S, et al (2000) A ribonucleotide reductase gene involved in a p53-dependent cell-cycle checkpoint for DNA damage. Nature 404: 42–49. 10716435 10.1038/35003506

[11] Zhou S, Zhao Y, He Y, Li H, Bulterys M, et al (2010) Hepatitis B and hepatitis C seroprevalence in children receiving antiretroviral therapy for human immunodeficiency virus-1 infection in China, 2005–2009. J Acquir Immune Defic Syndr 54: 191–196. 20032784 10.1097/QAI.0b013e3181c99226 PMC2877757

[12] Mambo E, Gao X, Cohen Y, Guo Z, Talalay P, et al (2003) Electrophile and oxidant damage of mitochondrial DNA leading to rapid evolution of homoplasmic mutations. Proc Natl Acad Sci U S A 100: 1838–1843. 12578990 10.1073/pnas.0437910100 PMC149920

[13] ter Hofstede HJ, Willems HL, Koopmans PP (2004) Serum L-lactate and pyruvate in HIV-infected patients with and without presumed NRTI-related adverse events compared to healthy volunteers. J Clin Virol 29: 44–50. 14675869 10.1016/s1386-6532(03)00085-4

[14] Gajewski CD, Yang L, Schon EA, Manfredi G (2003) New insights into the bioenergetics of mitochondrial disorders using intracellular ATP reporters. Mol Biol Cell 14: 3628–3635. 12972552 10.1091/mbc.E02-12-0796 PMC196555

[15] Wu Y, Li N, Zhang T, Wu H, Huang C, et al (2009) Mitochondrial DNA base excision repair and mitochondrial DNA mutation in human hepatic HuH-7 cells exposed to stavudine. Mutat Res 664: 28–38. 19428378 10.1016/j.mrfmmm.2009.02.006

[16] Wong LJ, Perng CL, Hsu CH, Bai RK, Schelley S, et al (2003) Compensatory amplification of mtDNA in a patient with a novel deletion/duplication and high mutant load. J Med Genet 40: e125. 14627692 10.1136/jmg.40.11.e125 PMC1735312

[17] Bourdon A, Minai L, Serre V, Jais JP, Sarzi E, et al (2007) Mutation of RRM2B, encoding p53-controlled ribonucleotide reductase (p53R2), causes severe mitochondrial DNA depletion. Nat Genet 39: 776–780. 17486094 10.1038/ng2040

[18] Kollberg G, Darin N, Benan K, Moslemi AR, Lindal S, et al (2009) A novel homozygous RRM2B missense mutation in association with severe mtDNA depletion. Neuromuscul Disord 19: 147–150. 19138848 10.1016/j.nmd.2008.11.014

[19] Nolan D, S Mallal (2004) Complications associated with NRTI therapy: update on clinical features and possible pathogenic mechanisms. Antivir Ther 9: 849–863. 15651744

[20] Barret B, Tardieu M, Rustin P, Lacroix C, Chabrol B, et al (2003) Persistent mitochondrial dysfunction in HIV-1-exposed but uninfected infants: clinical screening in a large prospective cohort. Aids 17: 1769–1785. 12891063 10.1097/00002030-200308150-00006

[21] Herman JS, Easterbrook PJ (2001) The metabolic toxicities of antiretroviral therapy. Int J STD AIDS 12: 555–562; quiz 563–564. 10.1258/0956462011923714 11516363

[22] Brinkman K, Kakuda TN (2000) Mitochondrial toxicity of nucleoside analogue reverse transcriptase inhibitors: a looming obstacle for long-term antiretroviral therapy? Curr Opin Infect Dis 13: 5–11. 11964766 10.1097/00001432-200002000-00002

[23] Yamanaka H, Gatanaga H, Kosalaraksa P, Matsuoka-Aizawa S, Takahashi T, et al (2007) Novel mutation of human DNA polymerase gamma associated with mitochondrial toxicity induced by anti-HIV treatment. J Infect Dis 195: 1419–1425. 17436221 10.1086/513872

[24] European Paediatric Lipodystrophy Group (2004) Antiretroviral therapy, fat redistribution and hyperlipidaemia in HIV-infected children in Europe. Aids 18: 1443–1451. 15199321 10.1097/01.aids.0000131334.38172.01

[25] Cossarizza A, Pinti M, Moretti L, Bricalli D, Bianchi R, et al (2002) Mitochondrial functionality and mitochondrial DNA content in lymphocytes of vertically infected human immunodeficiency virus-positive children with highly active antiretroviral therapy-related lipodystrophy. J Infect Dis 185: 299–305. 11807711 10.1086/338564

[26] Scruggs ER, Dirks Naylor AJ (2008) Mechanisms of zidovudine-induced mitochondrial toxicity and myopathy. Pharmacology 82: 83–88. 18504416 10.1159/000134943

[27] Wester CW, Okezie OA, Thomas AM, Bussmann H, Moyo S, et al (2007) Higher-than-expected rates of lactic acidosis among highly active antiretroviral therapy-treated women in Botswana: preliminary results from a large randomized clinical trial. J Acquir Immune Defic Syndr 46: 318–322. 18090299 10.1097/QAI.0b013e3181568e3f

[28] Papp E, Gadawski I, Côté HC (2008) Longitudinal effects of thymidine analogues on mtDNA, mtRNA and multidrug resistance (MDR-1) induction in cultured cells. J Antimicrob Chemother 61: 1048–1052. 18310046 10.1093/jac/dkn067

[29] Thelander L (2007) Ribonucleotide reductase and mitochondrial DNA synthesis. Nat Genet 39: 703–704. 17534360 10.1038/ng0607-703

[30] Sun R, Eriksson S, Wang L (2010) Identification and characterization of mitochondrial factors modulating thymidine kinase 2 activity. Nucleosides Nucleotides Nucleic Acids 29: 382–385. 20544523 10.1080/15257771003741018

[31] Vilà MR, Segovia-Silvestre T, Gámez J, Marina A, Naini AB, et al (2003) Reversion of mtDNA depletion in a patient with TK2 deficiency. Neurology 60: 1203–1205. 12682338 10.1212/01.wnl.0000055928.58122.47

[32] Pérez-Pérez MJ, Priego EM, Hernández AI, Familiar O, Camarasa MJ, et al (2008) Structure, physiological role, and specific inhibitors of human thymidine kinase 2 (TK2): present and future. Med Res Rev 28: 797–820. 18459168 10.1002/med.20124 PMC7168489

[33] Götz A, Isohanni P, Pihko H, Paetau A, Herva R, et al (2008) Thymidine kinase 2 defects can cause multi-tissue mtDNA depletion syndrome. Brain 131: 2841–2850. 18819985 10.1093/brain/awn236

